# A multimodal nomogram for benign-malignant discrimination of lung-RADS ≥4A nodules: integration of oxygen enhanced zero echo time MRI, CT radiomics, and clinical factors

**DOI:** 10.3389/fonc.2025.1563073

**Published:** 2025-07-21

**Authors:** Tiancai Yan, Ling Liu, Yuxin Li, Chunhui Qin, Haonan Guan, Tong Zhang

**Affiliations:** ^1^ Department of Radiology, The Fourth Hospital of Harbin Medical University, Harbin, China; ^2^ GE Healthcare, MR Research China, Beijing, China

**Keywords:** oxygen enhanced, CT, MRI, radiomics, multimodel analysis

## Abstract

**Background and objective:**

Lung-RADS ≥4A nodules require urgent intervention. Low-dose CT (LDCT), the primary screening tool, involves cumulative radiation exposure—critical for patients with serial scans. Oxygen-enhanced zero-echo time MRI (OE-ZTE-MRI) shows potential for lung nodule evaluation. However, its additive value when combined with CT radiomics and clinical factors for Lung-RADS ≥4A nodules remains unproven. This study aimed to develop a preoperative prediction model integrating OE-ZTE-MRI/CT radiomics and clinical factors for benign-malignant discrimination of Lung-RADS ≥4A nodules and compare its performance against single-modality models.

**Methods:**

99 nodules from 84 prospectively enrolled patients undergoing both LDCT and OE-ZTE-MRI were included. Nodule boundaries were manually contoured as regions of interest (ROIs) on both modalities. Six machine learning classifiers were applied to radiomic features (extracted from LDCT and OE-ZTE-MRI) and clinical parameters (age, smoking history, nodule diameter, calcification, etc.). Model performance was evaluated using receiver operating characteristic (ROC) curves with area under the curve (AUC), complemented by decision curve analysis (DCA). Univariate and multivariate logistic regression identified independent predictors, which were incorporated into a final nomogram to visualize clinical-radiomic prediction.

**Results:**

MRI model had a similar diagnostic performance to CT model (MRI *vs*. CT: training cohort AUC: 0.854 *vs* 0.907; testing cohort AUC: 0.769 *vs* 0.798). Multi-radiomics model achieved the highest diagnostic efficiency (train cohort AUC:0.923; testing cohort AUC: 0.813). Multivariate Logistic regression showed that nodule diameter (*p*=0.005) and calcification (*p*=0.029) were important factors affecting the benign and malignant nodules. The nomogram constructed by 3 models(CT/OE-ZTE-MRI/Clinical factors) achieved the best preoperative prediction performance for benign and malignant nodules (training cohort: AUC 0.941; testing cohort AUC:0.838).

**Conclusion:**

The nomogram combining OE-ZTE-MRI/CT radiomics and clinical factors (nodule diameter, calcification) improves preoperative discrimination of Lung-RADS ≥4A nodules (AUC=0.838), outperforming single-modality models. This tool enables evidence-based triage, potentially reducing unnecessary invasive procedures.

## Introduction

Lung cancer has become the leading cause of cancer-related deaths worldwide (1). Making clinical decisions for lung nodules of different risk levels as early as possible is an important means of preventing and treating cancer (2, 3). According to the Lung Imaging Reporting and Data System (Lung-RADS), solid nodules larger than 8mm or some part-solid nodules are defined as high-risk nodules (4)of 4A and above, requiring LDCT scans every three months or timely clinical intervention. However, continuous LDCT scans may increase radiation accumulation, leading to the malignancy of certain nodules. Pulmonary MRI scans have many advantages, including no ionizing radiation (5, 6), suitability for follow-up (7), and low noise (8).

Compared to traditional MRI imaging, UTE and ZTE (9, 10) aim to eliminate artifacts as much as possible with TE less than 1ms or even shorter, quickly converting quantitative signal parameters into easily distinguishable images (8, 9). A recent study demonstrated that ZTE sequences provide superior signal-to-noise ratio (SNR) and contrast-to-noise ratio (CNR) compared to UTE for pulmonary nodule detection (8). In addition, some reports (10, 11)have demonstrated that the use of ZTE-MRI has similar diagnostic performance to CT in Lung-RADS grading or lung density. Radiomics—systematically extracting quantitative imaging features from lesion and perilesional regions using automated, high-throughput computational algorithms—enables non-invasive characterization by correlating these features with clinical/pathological outcomes to identify optimal biomarkers for lesion evaluation (12). The combination of UTE or ZTE in Oxygen Enhancement (OE) technology successfully extracted and compared radiomics features from lung OE-UTE-MRI images and CT images (13). Some scholars have combined OE-UTE-MRI with quantitative MRI sequences and successfully identified the invasiveness of certain lung cancers (14).

In recent years, researchers have combined relevant radiomic parameters from CT and MRI to successfully predict postoperative adjuvant treatment patterns in gastric cancer (15). However, at the present stage, there are few studies combining OE-ZTE-MRI with CT. Meanwhile, there is an urgent need to find a method to combine multimodal radiomics with clinical parameters to accurately predict the benignancy or malignancy of high-risk nodules before surgery.

Therefore, this study is to construct a multimodel using CT and OE-ZTE-MRI radiomics features with clinical factors to predict the benign and malignant nodules before surgery and improve the preoperative prediction accuracy of lung nodules. Additionally, this study compares the performance of the multimodal radiomics model with the single radiomics model in preoperative prediction.

## Materials and methods

This is a prospective study, and the hospital’s Ethics Review Committee approved all procedures. The study adheres to the principle of complete confidentiality of subject information, and all researchers have full control over the provided radiological data.

**Figure 1:**
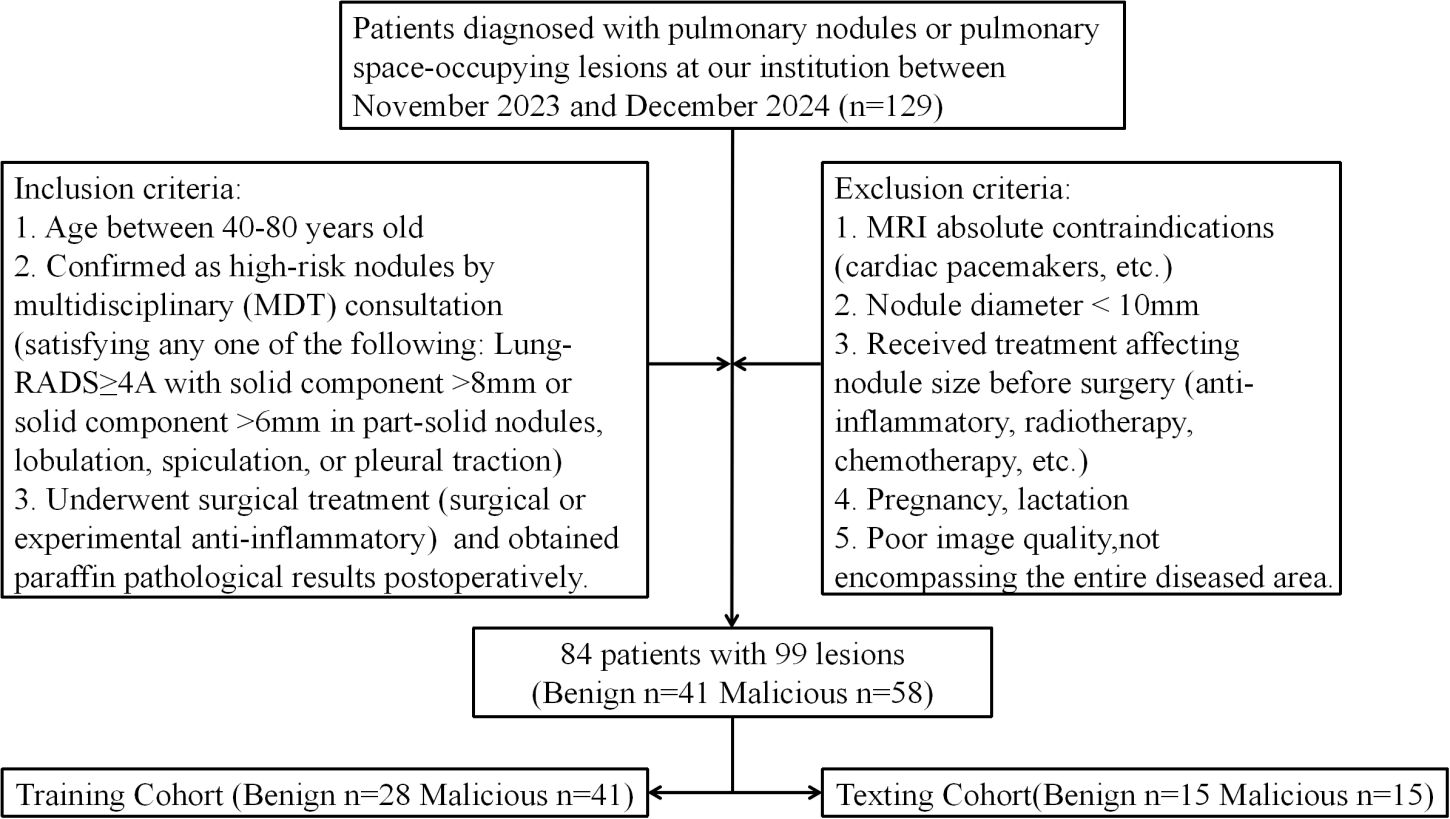
Flow chart of patient enrollment in this study.

The principles of ≪Declaration of Helsinki≫ conducting the study, and patients had full informed consent rights. From November 2023 to December 2024, patients diagnosed with “lung nodules” in the thoracic surgery department of our hospital were consecutively collected. These patients underwent chest CT and chest OE-ZTE-MRI examinations in our hospital and were preparing for surgical treatment. The inclusion criteria were: 1. Age between 40–80 years old; 2. Confirmed as high-risk nodules by multidisciplinary (MDT) consultation (satisfying any one of the following: Lung-RADS≥4A with solid component >8mm or solid component >6mm in part-solid nodules, lobulation, spiculation, or pleural traction); 3. Underwent treatment (surgical or experimental anti-inflammatory) and obtained paraffin pathological results postoperatively. The exclusion criteria were: 1. MRI absolute contraindications (cardiac pacemakers, etc.); 2. Nodule diameter < 10mm; 3. Received treatment affecting nodule size before surgery (anti-inflammatory, radiotherapy, chemotherapy, etc.); 4. Pregnancy, lactation;5. Poor image quality, not encompassing the entire diseased area. Huang et al.’s (16) result showed that UTE sequences achieve 100% detection sensitivity for pulmonary nodules ≥10 mm. This study excluded nodules <10 mm in diameter to ensure complete OE-ZTE-MRI visualization and accurate same-session CT co-registration for all enrolled lesions. According to the expert consensus in some Asian countries, it is recommended to conduct lung cancer screening for people over 40 years old with high-risk factors (17). And to comprehensively consider factors such as the physical functions of the elderly population, we have set the upper age limit at 80 years old. For some patients, there were multiple nodules in their lungs. These patients could obtain pathological results separately through surgical resection or CT-guided puncture. Each nodule’s pathological result was considered an independent diagnostic result. Based on the complete pathological results of this study, the outcome events were divided into benign and malignant groups. **Figure 1** illustrates the patient selection process and exclusion criteria. The dataset of this study was randomly divided into training and test sets in a 7:3 ratio.

All patient information and scan data from the Picture Archiving and Communication System (PACS) were anonymized to obtain the final imaging results and saved accordingly. Clinical information of patients was collected through the electronic medical record system, including: 1) Age; 2) Gender; 3) Maximum nodule diameter; 4) Body Mass Index (BMI); 5) Hypertension; 6) Diabetes; 7) Coronary Heart Disease (CHD); 8) Smoking history (18–21). All relevant demographic data and related information are summarized in **Table 1**. The nodules were independently evaluated by two radiologists to ensure accuracy. All nodules were jointly evaluated by two radiologists with extensive experience to have at least one of lobulation, spiculation, pleural traction, or nodule composition (i.e., Lung-RADS≥4A), and were included in this study after screening.

**Table 1:** Clinical features of the patients.

Variables	Train cohort benign	Train cohort malignant	Test cohort benign	Test cohort malignant
Age, years, mean±SD	53.68±13.59	62.10±9.84	60.60±11.02	64.13±10.94
Diameter, mm, mean±SD	14.18±8.95	27.49±21.27	17.47±9.48	33.67±22.97
BMI, kg/m^2^, mean±SD	24.09±2.42	22.45±2.71	23.19±2.86	24.41±3.72
CT Value, HU, mean±SD	-88.73±336.58	-227.71±309.60	-57.49±159.46	-140.88±267.65
Sex				
Female, n (%)	16 (57.14)	24 (58.54)	7 (46.67)	9 (60.00)
Male, n (%)	12 (42.86)	17 (41.46)	8 (53.33)	6 (40.00)
Density				
SN, n (%)	17 (60.71)	22 (53.66)	12 (80.00)	6 (40.00)
PSN, n (%)	7 (25.00)	9 (21.95)	2 (13.33)	8 (53.33)
GGN, n (%)	4 (14.29)	10 (24.39)	1 (6.67)	1 (6.67)
Location				
LAL, n (%)	9 (32.14)	14 (34.15)	2 (13.33)	1 (6.67)
LUL, n (%)	6 (21.43)	4 (9.76)	3 (20.00)	3 (20.00)
RAL, n (%)	5 (17.86)	14 (34.15)	6 (40.00)	6 (40.00)
RML, n (%)	3 (10.71)	1 (2.44)	2 (13.33)	1 (6.67)
RUL, n (%)	5 (17.86)	8 (19.51)	2 (13.33)	4 (26.67)
Calcification				
None, n (%)	22 (78.57)	40 (97.56)	13 (86.67)	13 (86.67)
Yes, n (%)	6 (21.43)	1 (2.44)	2 (13.33)	2 (13.33)
Hypertension				
None, n (%)	20 (71.43)	25 (60.98)	9 (60.00)	9 (60.00)
Yes, n (%)	8 (28.57)	16 (39.02)	6 (40.00)	6 (40.00)
Diabetes				
None, n (%)	26 (92.86)	37 (90.24)	11 (73.33)	13 (86.67)
Yes, n (%)	2 (7.14)	4 (9.76)	4 (26.67)	2 (13.33)
Coronary_Heart_Disease				
None, n (%)	23 (82.14)	33 (80.49)	13 (86.67)	11 (73.33)
Yes, n (%)	5 (17.86)	8 (19.51)	2 (13.33)	4 (26.67)
Smoking				
None, n (%)	16 (57.14)	26 (63.41)	7 (46.67)	12 (80.00)
Yes, n (%)	12 (42.86)	15 (36.59)	8 (53.33)	3 (20.00)

Values are mean ± SD, median ± 25th and 75th percentile, or n (%). P values signify statistical significance and reflect the differences between the Train and Text groups. SD, standard deviation; BMI, body mass index; SN, Solid Nodule; PSN, Part Solid Nodule; GGN, Gurond Glass Nodule; LAL, Left Anterior Lung; LUL, Left Under Lung; RAL, Right Anterior Lung; RML, Right Middle Lung; RUL, Right Under Lung; CHD, Coronary Heart Disease.

### MRI scanning protocol

All patient MRI scans were performed using a 3-T MRI device (Signa Premier, GE Healthcare) with a 32-channel spine coil and a 16-channel abdominal coil. The scanning parameters were as follows: repetition time/echo time: 770.4 ms/0.02 ms; field of view: 420 × 420 mm²; slice thickness: 2 mm; flip angle: 2°; voxel size:1.4×1.4×2.0; bandwidth: 45.46 kHz; flip angle: 2°; number of slices: 80-120 (depending on patient height).

The average acquisition time using OE-ZTE-MRI technology was 290 seconds (range: 285–407 s). Before starting the MRI scan, patients were manually instructed to inhale oxygen for two minutes. Subsequently, patients breathed nominally 100% oxygen (i.e., OE-ZTE-MRI sequence). The total MRI scan time did not exceed 7 minutes.

### CT scanning protocol

All patients underwent CT scans using 256-slice or higher CT scanners to obtain images and were diagnosed with “lung nodules” by two radiologists. During the scan, patients were scanned from the thoracic inlet, starting at the suprasternal notch, to the costophrenic angle. The scanning parameters were as follows: field of view: 256×256 mm² or 332×332 mm²; tube current: automatic; tube voltage: automatic (using automatic exposure control, depending on the patient’s weight). Images were reconstructed using standard mode and a slice thickness of 1-1.25 mm, and only preoperative images were used for image analysis. All CT results are plain scans, and contrast agents were not used.

### Feature extraction, selection, model construction, and statistical analysis

This study selected axial chest CT and OE-ZTE-MRI for feature extraction. We only chose T↓2-weighted images from the OE-ZTE-MRI sequence to compare with CT images. This choice was based on the findings of Ohno et al (22).

The DICOM medical digital images from CT and OE-ZTE-MRI were imported into the open-source software ITK-snap, and ROIs were delineated layer by layer using manual or semi-automatic methods. The tumor margins were delineated on CT images with a window width of 1600 HU and a window level of -600 HU and manually delineated layer by layer on OE-ZTE-MRI images with appropriate contrast. A radiologist with 20 years of experience in chest imaging diagnosis will carefully consider the relationship between the nodule and blood vessels or bronchi. According to the standardized CT imaging protocol, the radiologist will distinguish the nodule from the surrounding normal tissues. Meanwhile, under different imaging modalities, the radiologist will segment the pulmonary nodules based on factors such as the density of the tumor or the signal intensity on MRI images to ensure the rigor of nodule segmentation. Image segmentation was independently performed by two experienced radiologists who were blinded to the patients’ histopathology.

Since CT and OE-ZTE-MRI are different scanners with different data acquisition protocols, the data from these sequences usually have heterogeneous voxel spacing. It is necessary to preprocess these images. In this heterogeneous voxel spacing, we referred to the suggestions given in previous literature (23, 24). Spatial normalization was used to reduce the impact of voxel spacing variations, and fixed-resolution resampling was used to address the above issues. All images were resampled to a voxel size of 1×1×1 mm^3^ to standardize voxel spacing and standardized using z-score normalization (zero-mean normalization).

### Feature selection

The open-source installer named ‘PyRadiomics’ was used on Python to extract signal intensity features and texture features from the raw images. This software allows the resampling of both sets of sequence images to a voxel size of 1×1×1 mm. All the calculation formulas and the pipeline can be found at https://pyradiomics.readthedocs.io/en/latest/.iomic features. Z-score normalization was employed to address the issue of varying scales in manual radiomic features.

We performed a Mann-Whitney U test and feature screening for all radiomic features. Only the radiomic features with *p* < 0.05 were kept (230 CT features, 51 OE-ZTE-MRI features, and 281 CT-MRI features). For features with high repeatability, Pearson’s rank correlation coefficient was also used to calculate the correlation between features, and one of the features with a correlation coefficient greater than 0.9 between any two features is retained. After this, 67 CT features, 28 OE-ZTE-MRI features, and 50 CT-MRI features were kept. A Least Absolute Shrinkage and Selection Operator (LASSO) regression model was used for model construction on the discovery dataset. Depending on the tuning weight λ, LASSO shrinks all regression coefficients toward zero and sets the coefficients of many irrelevant features to zero. To find an optimal λ, a 10-fold cross-validation with the minimum standard was used, where the final value of λ produced the minimum cross-validation error. The regression model was fitted using the retained non-zero coefficient features and combined into a model. The Python scikit-learn package was used for LASSO regression modeling.

### Radiomics model construction

After LASSO feature selection, the final features were input into a Support Vector Machine (SVM) to construct the risk model. The following classifiers were used for machine learning, and a 5-fold cross-validation was employed to obtain the final radiomics model. Six machine learning classifiers with AUC > 0.69 were selected in descending order for this study. The ROC curve was plotted to evaluate the diagnostic performance of the prediction model, and the corresponding AUC value, sensitivity (SEN), specificity (SPE), positive predictive value (PPV), and negative predictive value (NPV) were analyzed. Decision curve analysis (DCA) was plotted to evaluate clinical effectiveness.

### Clinical model construction

Features for establishing the clinical model were selected through baseline demographic statistics. The same machine-learning models were used in the construction of the clinical model. A 5-fold cross-validation and test cohort were set up for fair comparison. Only the statistics screened by univariate and multivariate logistic regression models were finally included in the construction of the final model. Ultimately, the nomogram was constructed and compared with other models.

### Statistical analysis

Statistical analysis of radiomics was performed using Python and SPSS software packages. Shapiro-Wilk test was used to evaluate the normality of continuous data. Normally distributed continuous variables were expressed as mean ± standard deviation, while non-normally distributed variables were expressed as median and interquartile range. Categorical variables were analyzed using the chi-square test or Fisher’s test and expressed as absolute values (percentages). Paired AUC comparisons between classifiers were performed using DeLong’s test. Multivariate logistic regression analysis was used to determine statistically significant imaging parameters (two-tailed *p* < 0.05 was considered statistically significant) for inclusion in the multimodel. Area Under Curve (AUC) and accuracy (ACC) were calculated, along with their 95% confidence intervals. SEN, SPE, negative NPV, and PPV were also calculated. Hosmer-Lemeshow test was used to assess the goodness of fit. *p* < 0.05 was considered statistically significant.

## Results

### Baseline demographic characteristics

A total of 84 patients with 99 nodules clinically diagnosed with “pulmonary lesions” were included in this study. The average age of the patients was 59.80 ± 11.86 years, and **Table 1** shows other demographic data. Our statistical results showed no significant differences in high-risk factors such as age and gender between the two groups (*p* > 0.05). Among them, 41 nodules were benign, and 58 nodules were malignant. The majority of malignant nodules were adenocarcinomas (n=39,n=39.3%), squamous carcinomas (n=3,3.0%), and small-cell carcinomas (n=5,5.0%), and some of the malignant nodules were inoperable and were referred to conservative treatment (n=11,11.1%). Among benign nodules, there were some nodules with a clinical decision of benign and loss of pathological findings (n=17,17.1%), inflammatory nodules that shrank or disappeared after treatment (n=6,6%), alveolar epithelial hyperplasia (n=7,7%), misshapen tumors (n=4;4%), larger tuberculous foci (n=2,2%), benign mediastinal tumors (neurosphincteric tumors n=1;1% and benign cystic thymic adenoma n=1; 1%), adenomatoid hyperplasia (n=1,1%), mesenchymal tumors (n=1,1%) and sclerosing anaplastic tumors (n=1,1%).

**Figure 2:**
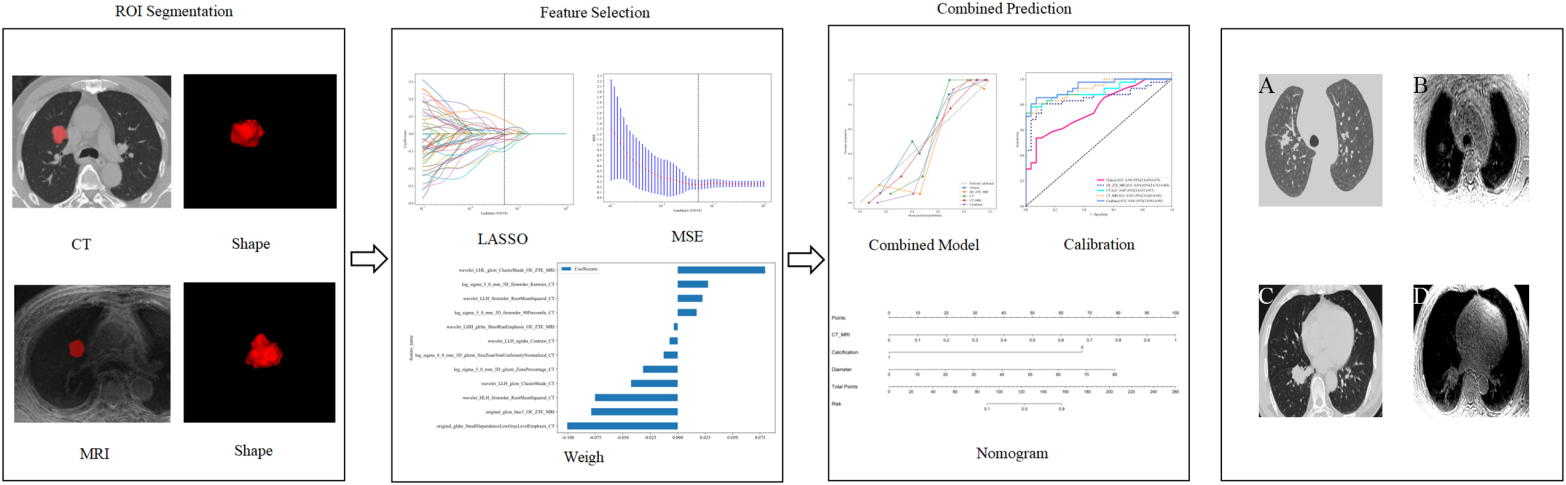
Workflow of radiomic analysis in the study. Radiologists segmented nodules, and LASSO selected features based on which the prediction model, MSE, and weight were built. Finally, the Combined Model, Calibration, and nomogram were built. Example figures **(A, C)** are CT images of the benign and malignant groups, respectively, while figures **(B, D)** are OE-ZTE-MRI images of the benign and malignant groups, respectively.

**Figure 3:**
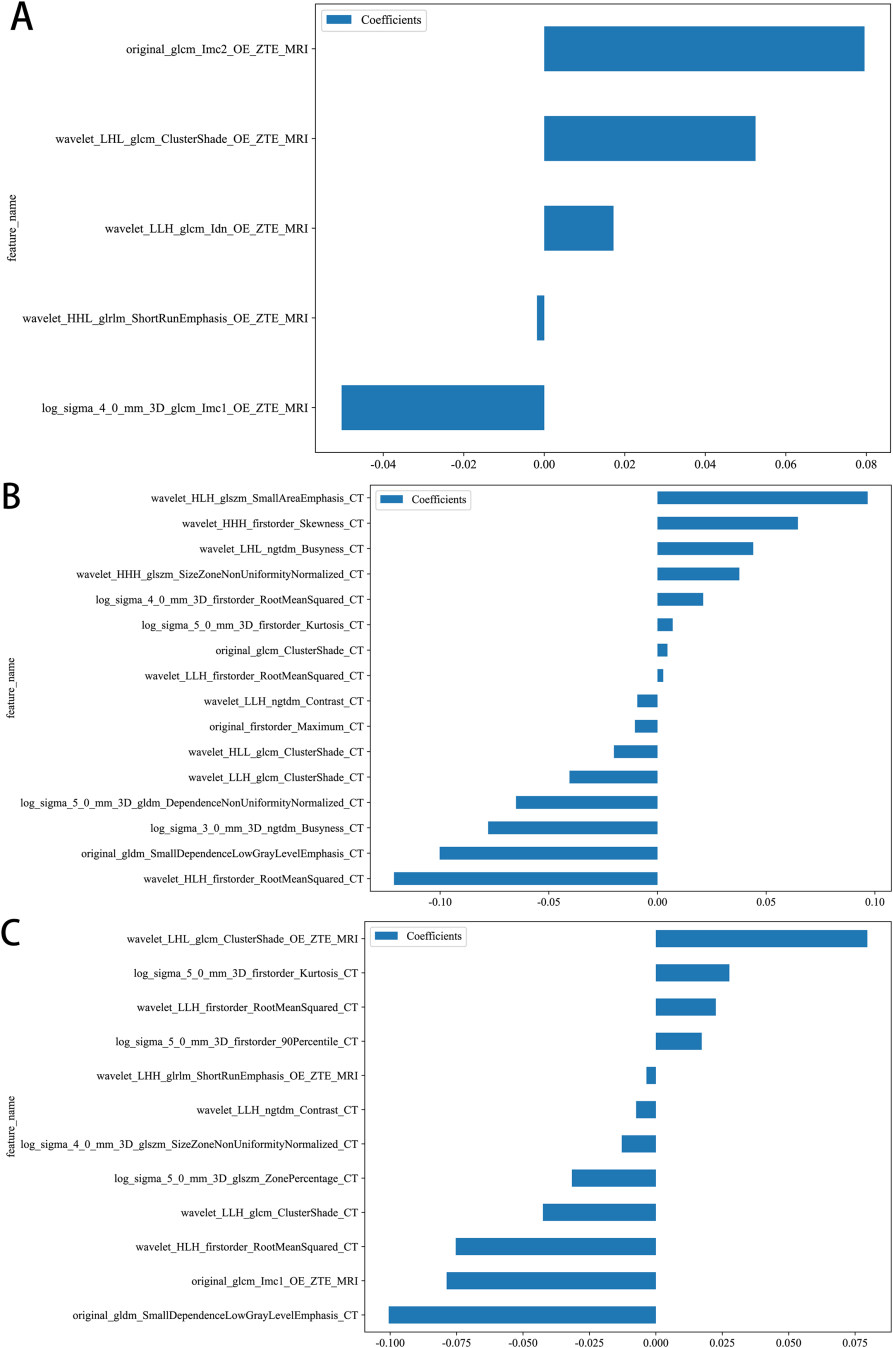
Retained radiomics features and their corresponding coefficients in different models after regression analysis by LASSO. **(A)** OE-ZTE-MRI model,**(B)** CT model, **(C)** CT-MRI model.

**Figure 2** shows the workflow of this study. A total of 2394 radiomics features (1197 per sequence, with the multi-radiomics model screening features from both sequences together) were extracted from CT and OE-ZTE-MRI images. The best features were input into the SVM model. The optimal model consisted of the following six models: 1. NativeBayes; 2. KNN; 3. Decision Tree; 4. LightGBM; 5. AdaBoost; 6. MLP. After three rounds of screening, 16 CT features, 5 OE-ZTE-MRI features, and 12 CT-MRI features were finally included in the analysis (**Figure 3**).

### Analysis of single and multi-radiomics model

**Figure 4:**
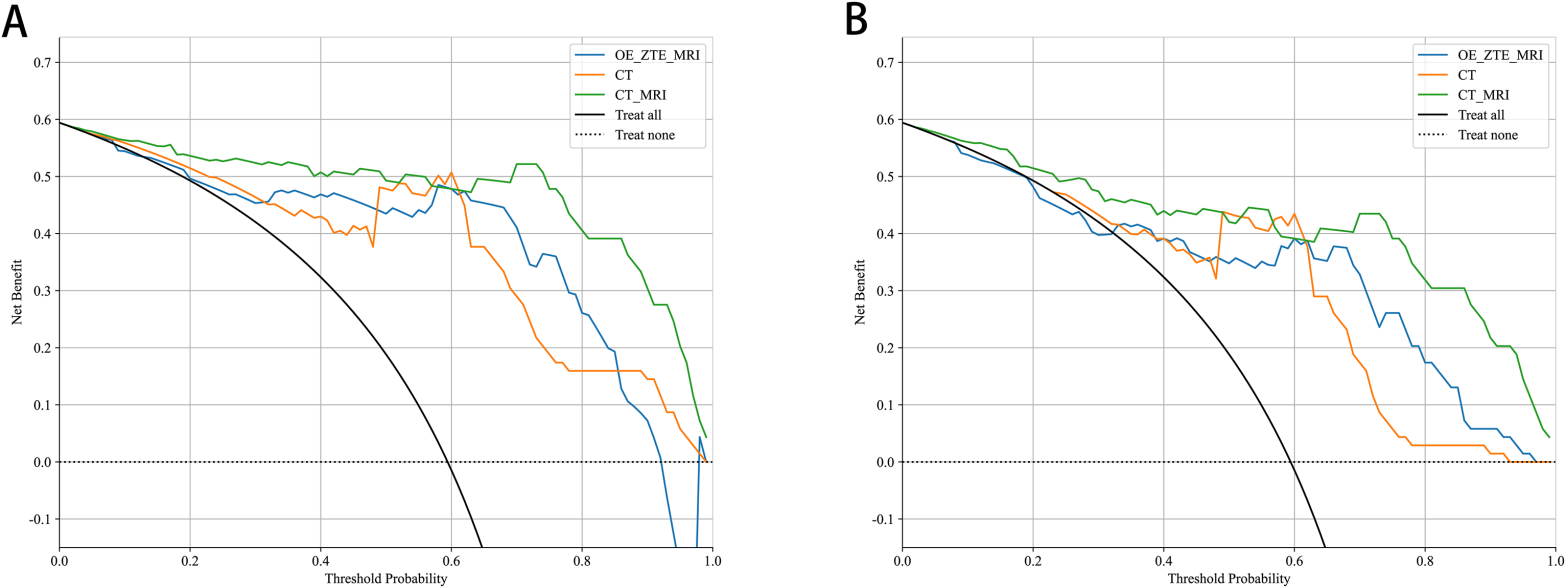
Decision Curve Analysis of MRI radiomic models. CT radiomic models and CT_MRI model for predicting Benign and Malicious in **(A)** training and **(B)** testing.

The selected radiomics features were used to construct six machine-learning models with the most suitable algorithms. In the three study cohorts, the AUC values of the training sets for the CT, OE-ZTE-MRI, and multi-radiomics(CT-MRI) model were 0.907 (95% CI: 0.837-0.977), 0.854 (95% CI: 0.761-0.946), and 0.923 (95% CI: 0.865-0.982), respectively. The AUC values of the test sets were 0.798 (95% CI: 0.634-0.962), 0.769 (95% CI: 0.587-0.951), and 0.813 (95% CI: 0.645-0.982), respectively, as shown in **Table 2**. DeLong test showed that the performance of the multi-radiomics model was significantly better than that of the single radiomics models (multi-radiomics *vs* CT: training cohort p=0.045; testing cohort p=0.032)(multi-radiomics *vs* MRI: training cohort p<0.001; testing cohort p<0.001) in terms of ACC, SEN, SPE, PPV, and NPV values. Although multi-radiomics model(training set AUC:0.923; testing set AUC: 0.813)had better predictive performance, MRI model also demonstrated diagnostic capabilities similar to the CT model (MRI *vs*.CT: training cohort AUC: 0.854 *vs* 0.907; testing cohort AUC: 0.769 *vs* 0.798). In this study, each radiomics model was also evaluated using DCA. DCA analysis for the test and training cohorts of each radiomics model is shown in **Figure 4**.

**Table 2:** CT, OE-ZTE-MRI and CT-MRI histology-based machine learning modeling analysis.

Cohort	Model	AUC	AUC 95%CI	Acc	Acc 95%CI	Sen	Spe	PPV	NPV
Train	CT_MRI	0.923	0.8651 - 0.9816	0.841	0.7367 - 0.9155	0.78	0.929	0.941	0.743
Test	CT_MRI	0.813	0.6452 - 0.9815	0.767	0.4247 - 0.7753	0.8	0.733	0.75	0.786
Train	CT	0.907	0.8370 - 0.9766	0.841	0.7367 - 0.9155	0.756	0.964	0.969	0.73
CTest	CT	0.798	0.6339 - 0.9617	0.733	0.4980 - 0.8354	0.6	0.867	0.818	0.684
Train	OE_ZTE_MRI	0.854	0.7609 - 0.9464	0.826	0.6853 - 0.8799	0.78	0.893	0.914	0.735
Test	OE_ZTE_MRI	0.769	0.5869 - 0.9509	0.767	0.4247 - 0.7753	0.733	0.8	0.786	0.75

95%CI, 95 percent confidence interval.

### Analysis of multi-radiomics model and clinical model

To intuitively and effectively evaluate the incremental prognostic value of the radiomics model for clinical factors, we constructed a combined model on the test dataset. This multi-model method integrates the multi-radiomics model and clinical factors based on univariate and multivariate logistic regression analyses. The construction process of the clinical model is essentially the same as that of the radiomics model. According to the results shown in **Table 3**, the univariate logistic regression consists of 12 parameters. Multivariate logistic regression ultimately showed significant differences (p < 0.05) in calcification shown by CT (OR: 0.667, 95% CI: 0.493-0.902, p=0.029) and larger nodule diameter (OR: 1.009, 95% CI: 1.004-1.013, p=0.005) between the two cohorts. This indicates that the risk of nodule malignancy is influenced by the nodule diameter and the presence of calcification within the nodule. Therefore, the selected clinical feature model consists of these two factors.

**Table 3:** A univariate and multivariate logistic regression analysis for clinical factors.

	Univariate Regression		Multivariate Regression	
Variable	OR (95%Cl)	p_value	OR (95%Cl)	p_value
Calcification	0.605 (0.441-0.829)	0.01	0.667 (0.493-0.902)	0.029
Smoking	0.938 (0.765-1.151)	0.606		
BMI	0.947 (0.914-0.981)	0.012	0.971 (0.939-1.003)	0.135
Sex	0.986 (0.806-1.208)	0.91		
CT_Value	1.000 (0.999-1.000)	0.082		
Location	1.003 (0.937-1.074)	0.941		
Diameter	1.010 (1.004-1.015)	0.003	1.009 (1.004-1.013)	0.005
Age	1.014 (1.006-1.022)	0.004	1.008 (1.001-1.016)	0.07
CHD	1.026 (0.795-1.326)	0.865		
Density	1.067 (0.942-1.209)	0.388		
Diabetes	1.083 (0.760-1.543)	0.71		
Hypertension	1.118 (0.907-1.377)	0.378		

OR (95%CI), Odds Ratio (95% Confidence Interval).

### Analysis of multi-model

**Figure 5:**
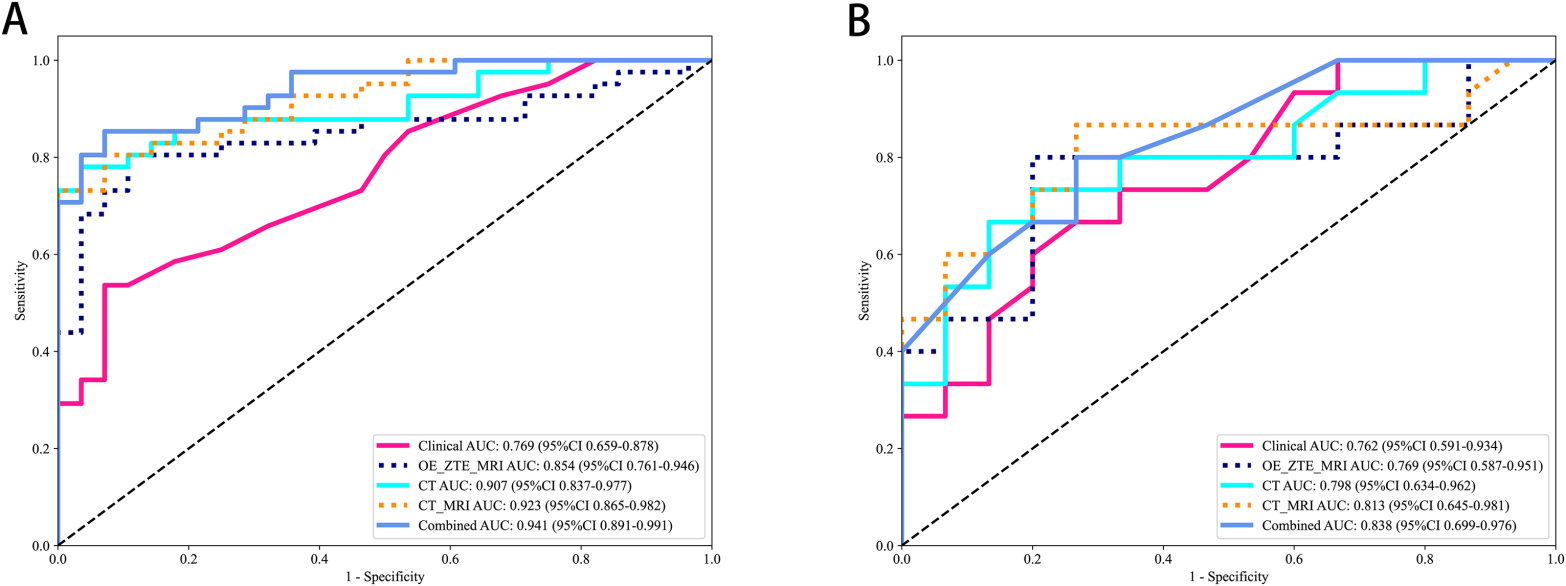
ROC curves of 5 models in the **(A)** training and **(B)** testing.

**Figure 6:**
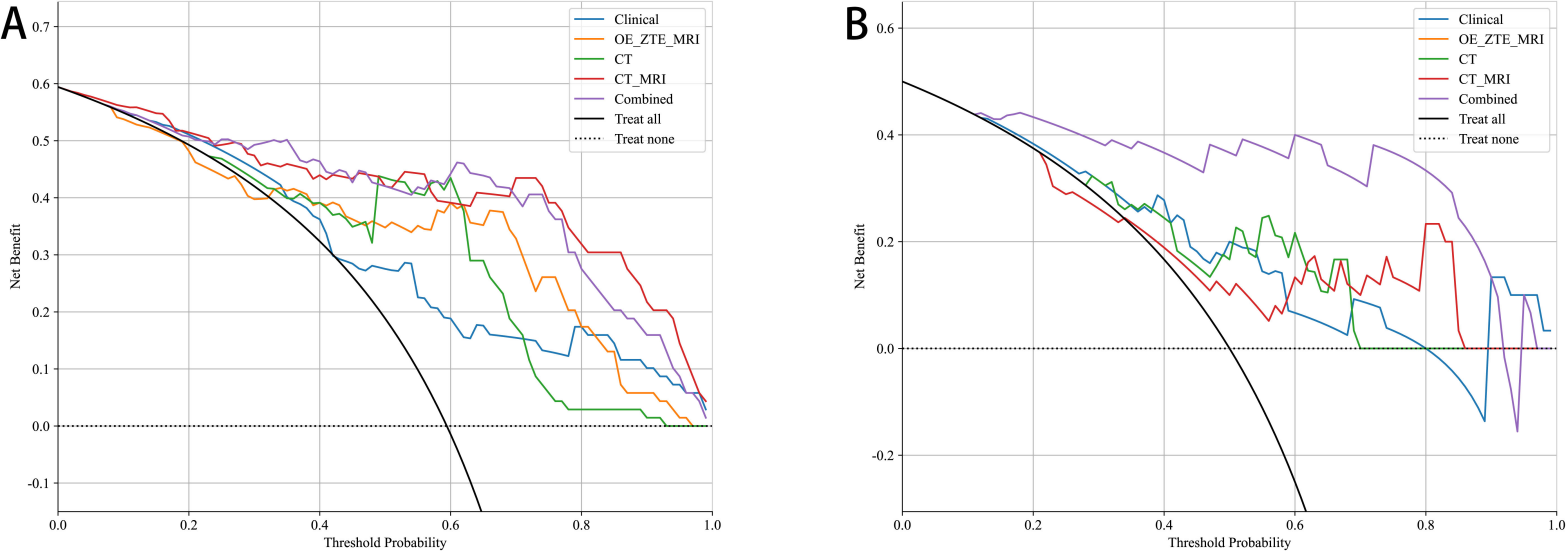
Decision Curve Analysis of 5 models for predicting Benign and Malicious in **(A)** training and **(B)** testing.

**Figure 7:**
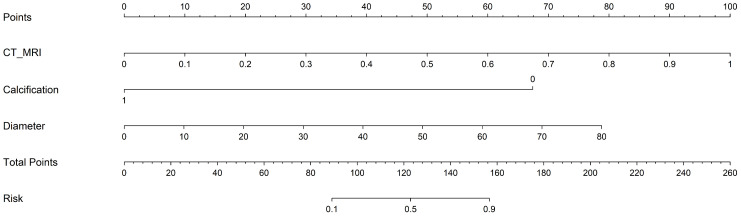
Nomogram of Combined Model. The sum of all factors equals the total points.CT_MRI: CT &OE-ZTE-MRI. Combined Model: CT sequences, OE-ZTE-MRI sequences, calcification and age. Among them, the nodule diameter is based on a zero baseline of 10mm.

**Figure 8:**
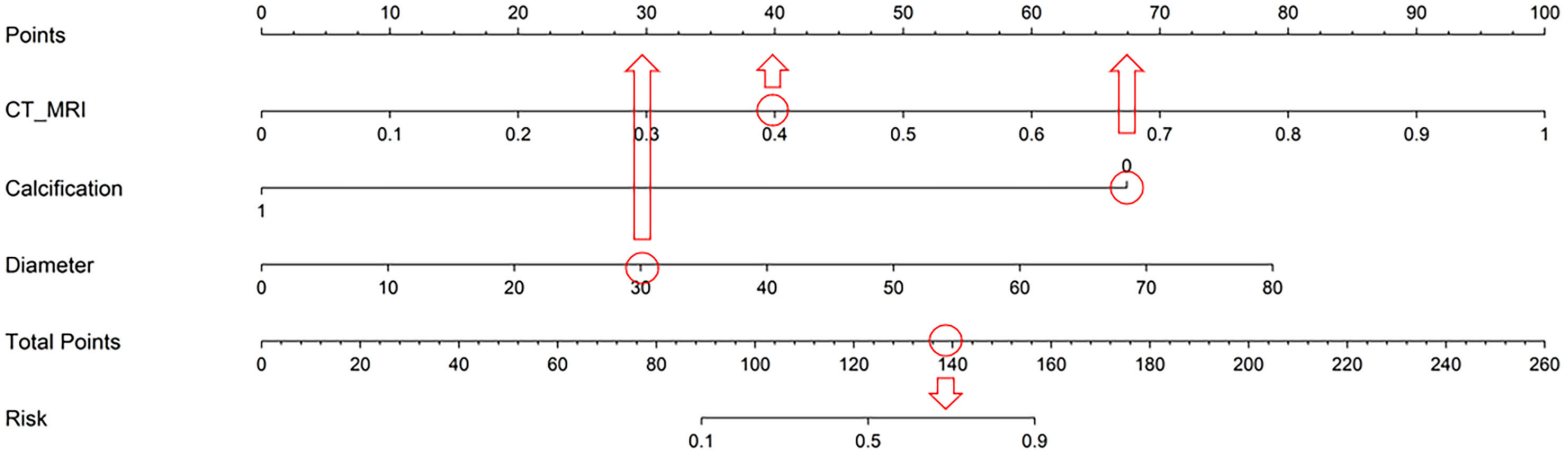
An example of a Nomogram of Combined Model. The CT-MR model predicted a 40% probability of malignancy in a patient with a pulmonary lesion. The nodule has no calcification and a diameter of approximately 40mm. After analysis using the nomogram, the malignancy rate for this patient increased to about 70%.

A combined model was constructed by integrating all radiomics models with clinical models (training cohort: AUC=0.941; testing cohort: AUC=0.838) (**Figure 5**). DeLong test showed that the multi-model outperforms the multi-radiomics model and the single clinical feature model(multi-model *vs* clinical: training cohort p<0.001; testing cohort p=0.001) (multi-model *vs* multi-radiomics model: training cohort p=0.035; testing cohort p=0.046). Hosmer-Lemeshow test indicated that the model had a good fit for predicting malignancy in both the training and testing cohorts (training cohort p=0.261; testing cohort p=0.163), with no significant difference between the predicted and actual values. The DCA results are shown in **Figure 6**. Compared to clinical models, the nomogram (**Figure 7**) and the example of the nomogram (**Figure 8**) provided higher net benefits, demonstrating greater potential valuation in clinical decision-making.

## Discussion

In this study, to preoperatively diagnose the malignancy of pulmonary nodules higher than Lung-RADS 4A, we developed and validated a new model. Based on this model, we generated a nomogram that can assist in clinical decision-making. This predictive model includes statistically significant clinical features, CT, and OE-ZTE-MRI radiomics features. Through comparison, we found that the multi-model constructed from clinical features and the CT-MRI model has greater advantages in preoperative diagnosis of the malignancy of such pulmonary nodules.

Gas MRI technology has been widely used in preclinical research. Currently, the commonly used gas contrast (25–27) agents, Helium-3 (^3^He) and Xenon-129 (^129^Xe), are difficult to prepare (28). Oxygen, due to its safety, may become the superior method for routine clinical operations using oxygen-enhanced MRI imaging schemes (29). Ohno et al. explored the feasibility of OE-MRI imaging in diagnosing lung diseases in clinical settings and compared the images of lung cancer patients with different tests, showing changes in regional oxygen concentration and lung function (22).

In recent decades, radiomics has rapidly developed, enabling the quantitative analysis of thousands of radiomics features. This helps assess lung tumors’ invasiveness and treatment response and assists in the preoperative differentiation of benign and malignant pulmonary nodules (30). Liu’s study (31) indicated that a combined model using radiomics and CT imaging features can differentiate between benign and malignant subcentimeter (≤10 mm) solid nodules (training cohort AUC: 0.942; testing cohort AUC: 0.930), which is almost consistent with the results obtained in this study using CT radiomics parameters alone (training cohort AUC: 0.907; testing cohort AUC: 0.798). However, their study did not target patients with non-solid nodules, which may have led to deviations in the AUC values in the test cohort. Additionally, it only differentiated between benign and malignant nodules without incorporating any clinical or imaging parameters. Huang’s results (32) showed that using a deep learning-enhanced radiomics model can successfully predict benign and malignant ground-glass nodules (GGNs), achieving the highest model performance in external validation. However, this study only targeted GGNs detected by CT, without including solid components, and was conducted in a single institution without external validation.

With the advent of UTE and ZTE technologies, it has become possible to use semi-automatic or manual delineation of tumor entities and extract their radiomics features. Wang (33) delineated multiparametric MRI sequences to extract radiomics features, successfully assessing the heterogeneity of lung tumors about nodule size. This study did not use UTE or ZTE sequences and did not further discuss benign nodules. UTE-MRI or ZTE-MRI, as described by the Fleischner Society (7), can be used as a supplementary sequence to help evaluate lung nodule imaging or perform feature extraction. Using ZTE is better than UTE, as validated by Bae’s (8) study. Compared to UTE, ZTE is superior in depicting normal lung structures. The shorter TE in ZTE may result in less signal loss at the nodule site, which is crucial for enhancing image clarity.

It is well known that CT and MRI are two technologies with completely different acquisition principles. Although GGNs and PGNs are less clear on MRI images (34), the cross-modal model of CT and OE-ZTE-MRI still achieved results similar to those of Lee (13), and higher than those reported by Wielputz (35). We speculate that during the patient selection process, the sizes of PGNs and GGNs were both over 10 mm, with nodule characteristics dominated by ground-glass components, and pathological results confirmed that almost all were malignant. Additionally, we found that radiomics features stable across modalities are not rare, and previous studies have demonstrated that some multimodal models can be applied in clinical practice (36). However, based on previous conclusions, we selected the most suitable texture features (13, 23)and constructed the optimal multimodal radiomics model. This has been validated in preclinical efficacy evaluations, such as a study on the correlation between multimodal radiomics and pathology of thermal ablation lesions in rabbit lung VX2 tumor models (37); it has also been supported in clinical practice by a machine learning-based study using multimodal radiomic texture features to predict lung cancer (38).

There are some limitations to this study. First, each subject had to undergo complete CT scans, and OE-ZTE-MRI examinations. Some elderly patients with underlying diseases (unable to lie down for long periods) could not tolerate all the examinations well, leading to a smaller sample size that may be accompanied by unpredictable selection bias. Second, the single-center, small-sample design of this study may compromise model generalizability, necessitating further multicenter external validation—preferably across heterogeneous cohorts and cross-modality verification (e.g., PET-CT)—to confirm robustness against equipment parameters, reader expertise, and regional population differences. Finally, this model may not be suitable for patients with larger nodules, as large masses (such as massive hilar tumors with obstructive pneumonia) are often accompanied by emaciation or severe underlying diseases. These patients are often inoperable, and the probability of malignancy is almost 100%. Therefore, early detection and multidisciplinary team collaboration are crucial for developing appropriate treatment plans.

## Conclusion

This study constructed a multi-model by integrating clinical factors, CT, and OE-ZTE-MRI radiomics features. This model can be used to predict the malignancy of high-risk nodules preoperatively, thereby improving the accuracy of pulmonary nodule prediction. DeLong test showed that in preoperative prediction of the malignancy of high-risk pulmonary nodules, multi-model>multi-radiomics model>single model.

## Data Availability

The raw data supporting the conclusions of this article will be made available by the authors, without undue reservation.
